# ChAMP: updated methylation analysis pipeline for Illumina BeadChips

**DOI:** 10.1093/bioinformatics/btx513

**Published:** 2017-08-14

**Authors:** Yuan Tian, Tiffany J Morris, Amy P Webster, Zhen Yang, Stephan Beck, Andrew Feber, Andrew E Teschendorff

**Affiliations:** 1 CAS Key Lab of Computational Biology, CAS-MPG Partner Institute for Computational Biology, Shanghai Institute for Biological Sciences, University of Chinese Academy of Science, Chinese Academy of Sciences, Shanghai, China; 2 Medical Genomics Group, Paul O’Gorman Building, UCL Cancer Institute, University College London, London, UK; 3 Cambridge Epigenetix, Jonas Webb Building, Babraham Campus, Cambridge, UK; 4 Statistical Genomics, UCL Cancer Institute, University College London, London, UK; 5 Department of Women’s Cancer, University College London, London, UK

## Abstract

**Summary:**

The Illumina Infinium HumanMethylationEPIC BeadChip is the new platform for high-throughput DNA methylation analysis, effectively doubling the coverage compared to the older 450 K array. Here we present a significantly updated and improved version of the Bioconductor package ChAMP, which can be used to analyze EPIC and 450k data. Many enhanced functionalities have been added, including correction for cell-type heterogeneity, network analysis and a series of interactive graphical user interfaces.

**Availability and implementation:**

*ChAMP* is a BioC package available from https://bioconductor.org/packages/release/bioc/html/ChAMP.html.

**Supplementary information:**

Supplementary data are available at *Bioinformatics* online.

## 1 Introduction

DNA methylation is the most studied epigenetic modification. Illumina’s new EPIC BeadChip can measure methylation at over 850 000 sites with single-nucleotide resolution. The EPIC BeadChip includes over 90% of probes present on the 450 K array, shows high reproducibility, and will become a common tool for epigenome-wide association studies (Moran *et al.*, 2016).

ChAMP is an integrated analysis pipeline published in 2014 (Morris *et al.*, 2014), which includes functions for filtering low-quality probes, adjustment for Infinium I and Infinium II probe design, batch effect correction, detecting differentially methylated positions (DMPs), finding differentially methylated regions (DMRs) and detection of copy number aberrations (CNA).

The new version of ChAMP, extends and improves this analysis pipeline, adding novel and enhanced functionalities, including detection of differentially methylated genomic blocks (DMB), gene set enrichment analysis (GSEA), a method for correcting cell-type heterogeneity and detection of differentially methylated gene modules. Notably, the new package provides a series of web-based graphical user interfaces (GUIs), which facilitate analyses and enhance user-experience.

## 2 Description

ChAMP is an R package and currently requires R(≥3.4). ChAMP loads data from IDAT files using it’s novel loading function, or though minfi loading function (Aryee *et al.*, 2014). Probes can be filtered based on detection *P*-values, chromosomal location, presence of single nucleotide polymorphisms in the probe sequence (Zhou *et al.*, 2016) and cross-hybridization. Multi-dimensional scaling, density and clustering plots allow exploratory analysis. For normalization, functional normalization (Fortin *et al.*, 2014) has been added as an option alongside beta-mixture quantile normalization (Teschendorff *et al.*, 2013). Singular value decomposition is used to correlate principal components to biological and technical factors, helping the user decide if there are batch effects or confounding factors that need to be adjusted for.

**Fig. 1:**
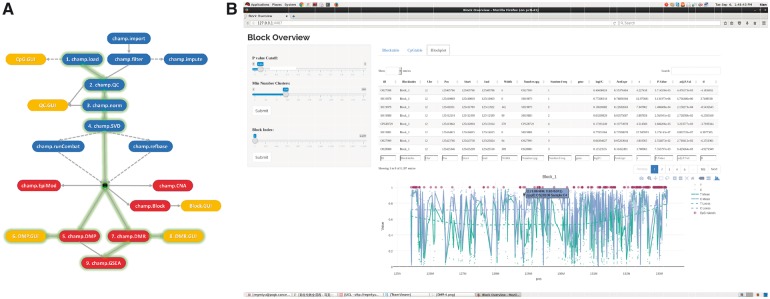
The ChAMP pipeline. **(A)** All functions included in ChAMP. Blue functions used for data preparation. Red functions used to generate analysis results. Yellow functions are GUI functions for visualization. Functions and edges with light green gleam stands for main pipeline (markers are steps for using ChAMP). Dash lines mean functions may not necessarily required. **(B)** GUI function for visualization of a DMB. The left panel displays parameters for controlling the plot and the table

For supervised analysis, besides limma-based DMP and ProbeLasso-based DMR analysis functions (Butcher and Beck, 2015), there is now added functionality for DMR detection using Bumphunter (Jaffe *et al.*, 2012) and DMRcate (Peters *et al.*, 2015). Large-scale differentially methylated blocks (DMB) can also be identified. These DMBs are large-scale genomic regions (10 kb–Mb) containing hundreds of inter-genic CpG sites (Fig. 1B), and which often exhibit hypomethylation in aging and cancer (Yuan *et al.*, 2015). We also added functionality to allow users to detect differentially methylated hotspots in user-defined gene networks (Jiao *et al.*, 2014). In addition, ChAMP incorporates GSEA capability on DMP and DMR results (Young *et al.*, 2010).

In ChAMP, correction for cell-type heterogeneity in blood can be performed with the reference-based RefbaseEWAS (Houseman *et al.*, 2012). Another unique feature of ChAMP is a function for detecting CNA (Feber *et al.*, 2014). As a result of all these functionalities, ChAMP is now a much more powerful and comprehensive tool for DNA methylation analysis (Fig. 1A).

Besides making all above functions applicable to EPIC BeadChips, there are two other technical improvements which will benefit users. First, ChAMP accepts multiple data input formats, including IDATS, beta-valued matrices and phenotype data files. Second, a series of javascript-based GUIs are provided. This allows easy checking of results, and generating figures for DMR or DMBs. Shiny, a web application framework for R, suitable for creating simple interactive webpages, and Plotly, an open source JavaScript graphing library, are integrated with ChAMP results, allowing users to view, select, and zoom in and out from results obtained with ChAMP. All GUIs use the results of ChAMP functions as parameters (Fig. 1B).

Full details and an example workflow of ChAMP are provided (Supplementary Material).

## 3 Conclusion

In summary, ChAMP provides a much improved, powerful and comprehensive pipeline for Illumina HumanMethylation BeadChip analysis.

## Funding

Royal Society and Chinese Academy of Sciences (Newton Advanced Fellowship 164914) [to A.E.T.]; Chinese Scholarship Council (CSC) [to Y.T.]; MRC [MR/M025411/1 to A.F.] and the UCLH/UCL Comprehensive Biomedical Research Centre [to A.F.]; and National Institute for Health Research (NIHR) Blood & Transplant Research Unit (BTRU) [NIHR-BTRU-2014-10074 to A.P.W. and S.B.].

*Conflict of Interest*: none declared.

## Supplementary Material

Supplementary Data
