# Supplementary material for: ChAMP: updated methylation analysis pipeline for Illumina BeadChips
Source: Bioinformatics. 2017 Aug 14;33(24):3982–4. doi: 10.1093/bioinformatics/btx513 (PMC5860089; doi:10.1093/bioinformatics/btx513)
Supplement: Supplementary Data [file btx513_supplementary.docx]

Supplementary for ChAMP: Updated Illumina Chip Analysis Methylation Pipeline

**Yuan Tian^1,2^, Tiffany J Morris^3^ , Amy P Webster^2^  , Zhen Yang^1^,**  **Stephan Beck^2, *^, Andrew Feber^2, *^, Andrew E Teschendorff ^1,3,4, *^**

1 CAS Key Lab of Computational Biology, CAS-MPG Partner Institute for Computational Biology, Shanghai Institute for Biological Sciences, Chinese Academy of Sciences, 320 Yue Yang Road, Shanghai 200031, China

2 Medical Genomics Group, Paul O’Gorman Building, UCL Cancer Institute, University College London, 72 Huntley Street, London WC1E 6BT, United Kingdom.

3 Cambridge Epigenetix, Jonas Webb Building, Babraham Campus, Cambridge CB22 3AT, UK.

4 Statistical Genomics Group, UCL Cancer Institute, University College London, 72 Huntley Street, London WC1E 6BT, United Kingdom.

5 Department of Women’s Cancer, University College London, 74 Huntley Street, London WC1E 6AU, United Kingdom.

ChAMP is a newly updated integrating analysis pipeline for DNA methylation bead chip data. Compared with the previous version, new version ChAMP adds functionality in terms of functions for cell-type heterogeneity correction, differential methylation block (DMB) analysis, Gene Set Enrichment Analysis (GSEA), functional epigenetic modules (FEM) analysis, as well as a series of graphical user interfaces based on the shiny and plotly packages. In the vignette of the package we illustrate the use of each function on a 450K dataset, while here we demonstrate the functionality of ChAMP on an EPIC dataset.

More information about processing this data set can be find in above link, we recoded all output from this EPIC dataset:

<https://github.com/JoshuaTian/ChAMPDemos/tree/master/DemoEPIC%20DataSet>

Specifically, we use the EPIC dataset GSE86831 (which can be downloaded from the GEO website). This set contains 15 samples, distributed across 4 phenotypes: a transformed prostate cancer cell line (LNCaP); primary cell cultures of prostate epithelial cells (PrEC); patient-matched cancer associated fibroblasts (CAF) and non-malignant tissue associated fibroblasts (NAF); infant blood from archival Guthrie cards.

We obtained the IDAT files for this dataset from the GEO website, and generated a sample sheet csv file containing sample information as shown below.


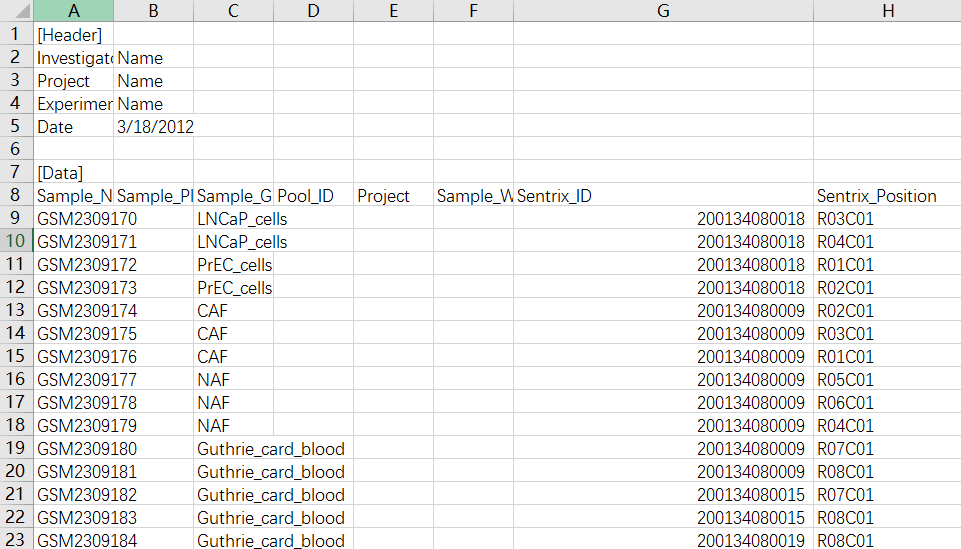


**Figure** 1: pd file for GSE86831. We generated this file by using GEOquery R package.

Before loading data with below code, users need to make sure the sample information csv file (like above one) is placed inside the same folder with IDAT file, because ChAMP will automatically detect samples from above csv file and load corresponding IDAT file.

myLoad <- champ.load(directory = "../Data",arraytype = "EPIC")

# loaded as minfi way

myLoad_2 <- champ.load("../Data/",method="minfi",arraytype="EPIC")

Now we provided two loading method, one is “minfi” way, which use minfi’s read.metharray.exp() function to load data from .idat file, this method would return rgSet and mset, which might be used in SWAN normalization, Functional Normalization and generating control probe in champ.SVD() function. Another method is new “ChAMP” way, which is a new loading function relying on champ.import() and champ.filter(), both are implemented by ChAMP team. So, if you want to use SWAN or FunctionalNormalization method, you may load data as “mnfi” way. You may specify parameter “method” in champ.load() function to choose method. In above code, myLoad is loaded as “ChAMP” way, and myLoad_2 is loaded as “minfi” way.

The loading function automatically reads all IDAT files into the R session and performs optional filtering of probes based on quality (i.e. using P-values of detection), on whether they map to sites on the X/Y chromosomes, and whether probes contain SNPs. The SNP list is provided by Zhou et al. NAR 2016. In this particular instance, champ.load(), with default filtering settings, results in 737950 CpG sites. Then we use QC.GUI() function to get an overview of the datasets, such as sample distribution, hierarchical clustering, Infinium I and Infinium II probe distribution .e.g.

QC.GUI(arraytype="EPIC")

This function plots density distributions of beta-values for all samples, and also separately for type-1 and type-2 probes, allowing problematic samples to be easily identified. In addition, it also performs multi-dimensional scaling and hierarchical clustering on the top 5% of probes selected from a Singular Value Decomposition of the data, allowing for exploratory analysis and to check for outliers.


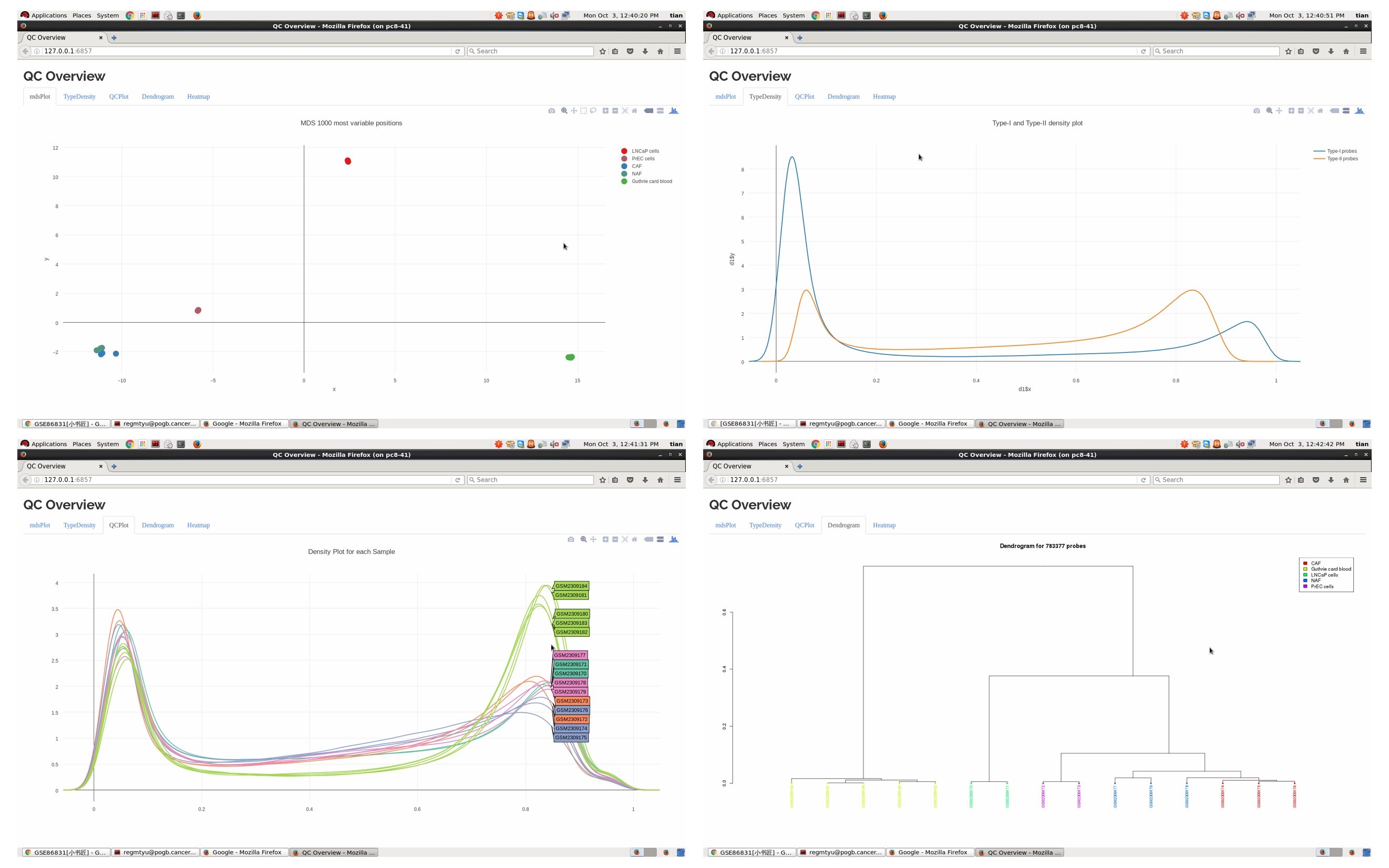


**Figure** 2: Plots generated by QC.GUI() function: In total, QC.GUI() generates 5 figures. We present 4 of them here. The mdsPlot (multi-dimensional scaling) is on the top-left corner, where each dot represents one sample. The top-right figure shows density distributions of Type-I and Type-II probes. The third plot on the bottom-left shows the beta-value density distributions of each sample. The last figure shows the clustering dendrogram of all samples.

According to the above plot, we can see that there is a significant shift between the type-I and type-II probes. To adjust for this, one can use the champ.norm() function, which can do the correction of the type-2 probe bias using a variety of different normalization methods. Here we use the BMIQ method.

myNorm <- champ.norm(arraytype="EPIC")

Note that ChAMP adds support for parallel computing, so it is possible to assign more cores to speed up this step. After BMIQ, the bias associated with type-2 probes is substantially reduced:


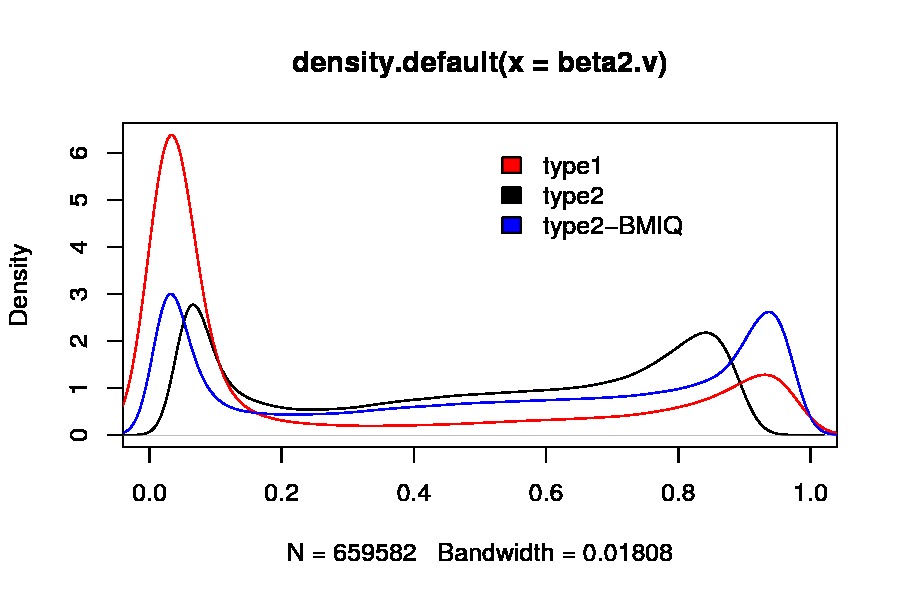


**Figure** 3**:** Normalization result of BMIQ, generated by champ.BMIQ() function. The red line indicates beta distribution for type-I probes, the black line indicates density plot for type-II probes, and the blue line indicates the normalized result for type-II probes.

Next step in the recommended analysis pipeline is to perform a Singular Value Decomposition (SVD) of the data, to assess what the top components of variation correlate with. Here we present two SVD plots, one which includes control probes (e.g. indicating effect of Red and Green channels), while the other does not:

# Change Slides parameter from numeric to character.

myLoad$pd$Slide <- as.character(myLoad$pd$Slide)

champ.SVD()

champ.SVD(RGEffect=T,rgSet=myLoad_2$rgSet)


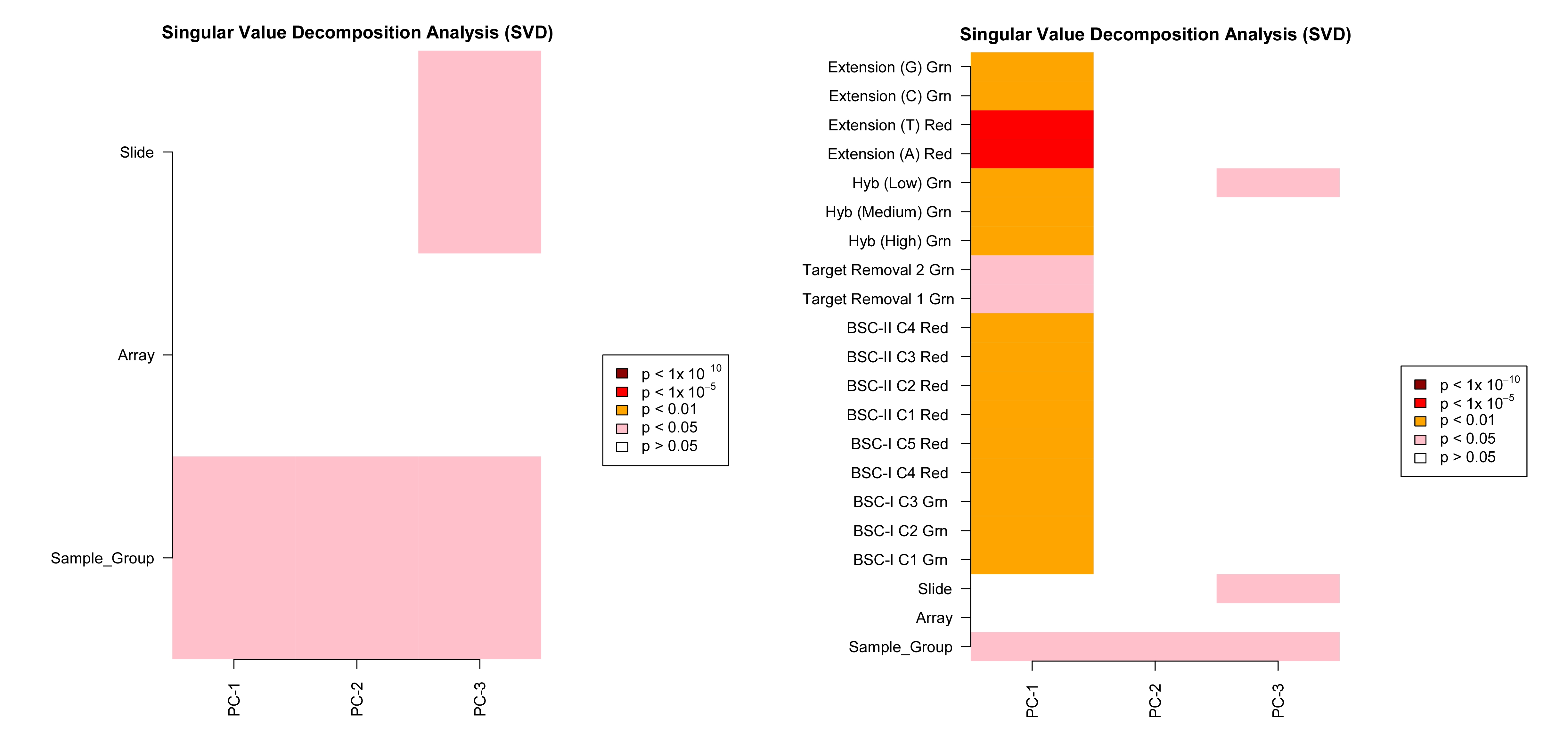


**Figure** 4**:** SVD analysis heatmap generated by champ.SVD() function. The left plot shows covariates/factors that exist in the pd file, while the right plot also includes control probes.

Note that for now control probe information (right panel) need to be extracted from rgSet. So you need to use “minfi” way to load data, get rgSet then put rgSet from champ.load() into champ.SVD(). In above code, we used myLoad_2$rgSet, because myLoad_2 is loaded from “minfi” way.

Based on the SVD heatmap, Sample_Group shows a significant correlation with the top 3 components, whilst “Slide” shows less influence on data variation. The P values are not very significant mainly because of the small sample size in this test dataset. For a data set, if there is strong evidence for confounding variation by Slide or Array, batch correction is possible with the champ.runCombat() function.

However champ.runCombat() is not suitable for this data set, because the “Slide” factor actually comes from Sentrix_ID column from pd.csv file at the beginning of this document. In Sentrix_ID, there are 4 phenotypes: 200134080018, 200134080009, 200134080015 and 200134080019. Combat algorithm can be applied on certain factor only of all phenotypes in that factor contains more than 2 samples. But phenotype 200134080019 contains only 1 phenotype. In this data set, the effect of Slide is not strong, so we can continue.

A typical next step in the analysis of a data set is to generate a list of differentially methylated probes (DMP), which with ChAMP we can do as follows:

> myDMP <- champ.DMP(arraytype = "EPIC")

In our new version champ.DMP() function, numeric variable is acceptable. If numeric variable is detected, linear regression would be conducted on each CpGs on this variable, to find linear-related CpGs. Also factors with multiple phenotypes are also acceptable, in old champ.DMP(), users may assign compare.group parameter to specify groups of samples they want to compare. In new version champ.DMP() if user leave compare.group as default NULL, champ.DMP() would do pairwise comparison on each pair of phenotypes. Here since our data sets contains multiple phenotypes, each pair of them would be calculated in champ.DMP().

After calculating DMP, a GUI function called DMP.GUI() is provided to show CpG information, draw gene and CpG plot. DMP.GUI() takes on data frame of champ.DMP() as input. So if you have multiple data frame result from champ.DMP() function, remember to specify the one you want to check into DMP.GUI() function. By default, DMP.GUI() would take the first data frame in myDMP list as input.
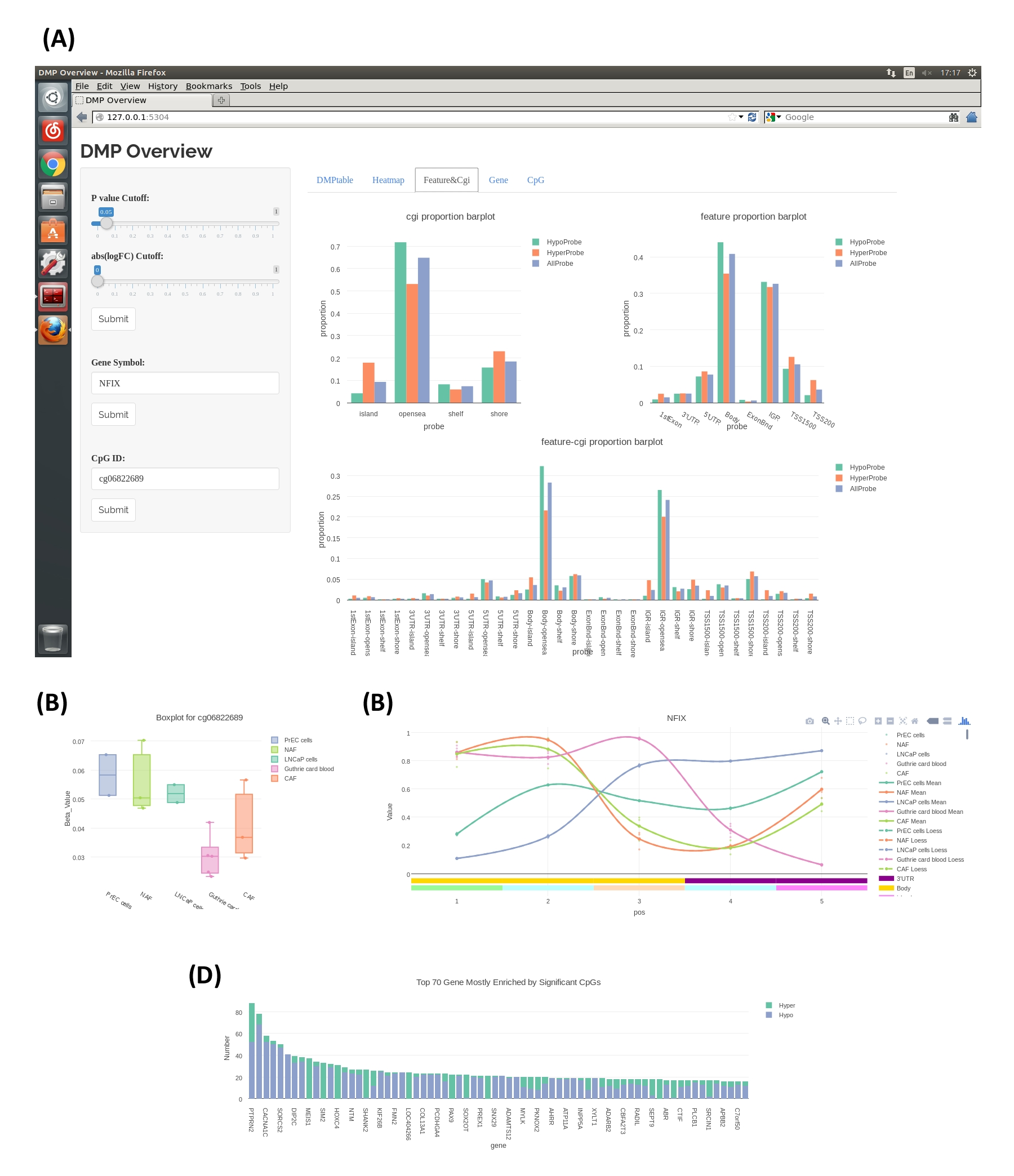


**Figure** 5**:** Plots drawn by DMP.GUI() function. **(A):** the distribution of DMPs across genomic features like CpG-island, opensea, 3’UTR, 5’UTR . **(B):** Boxplot of methylation values for selected CpGs. Boxplots can be closed by clicking the legend. **(C):** All significant CpGs enriched in gene NFIX. Solid lines indicate the mean value of each phenotype. **(D):** The enrichment status of top 70 most CpG-enriched genes. Hyper Methylated and Hypo Methylated are separated by different colors.

After DMP calling, we may wish to identify differentially methylated regions (DMRs). champ.DMR() offers the ability to detect DMRs using one of three different functions. Here we use DMRcate method to generate DMRs with code blow:

myDMR <- champ.DMR(arraytype = "EPIC",method="DMRcate",cores=1)

DMR.GUI(DMR=myDMR,arraytype="EPIC",compare.group=c("PrEC_cells","LNCaP_cells"))

After DMR detection, we can use the DMR.GUI() function to check the result of DMRs. Large DMRs (mega-base scale regions), called differentially methylated blocks (DMBs), can also be inferred and displayed using a GUI function. ChAMP detects DMBs by applying the bumphunter algorithm on collapsed small opensea clusters. Users can use following code to generate DMB:

myBlock <- champ.Block(arraytype = "EPIC")

Block.GUI(arraytype="EPIC",compare.group=c("PrEC_cells","LNCaP_cells"))

The interface for DMRs and DMBs are similar, both would present all DMRs and Blocks detected by ChAMP function, also nice plots for each region would be returned for downloading.

Note that in DMR.GUI() and Block.GUI(), user may set parameter “runDMP” to calculate p value for each CpG. For factors containing multiple phenotypes, you must specify which two groups you want to compare. For example in this data set, since Sample_Group in GSE86831 contains multiple phenotypes, we specified compare.group as c("PrEC_cells","LNCaP_cells") when running these two GUI functions.


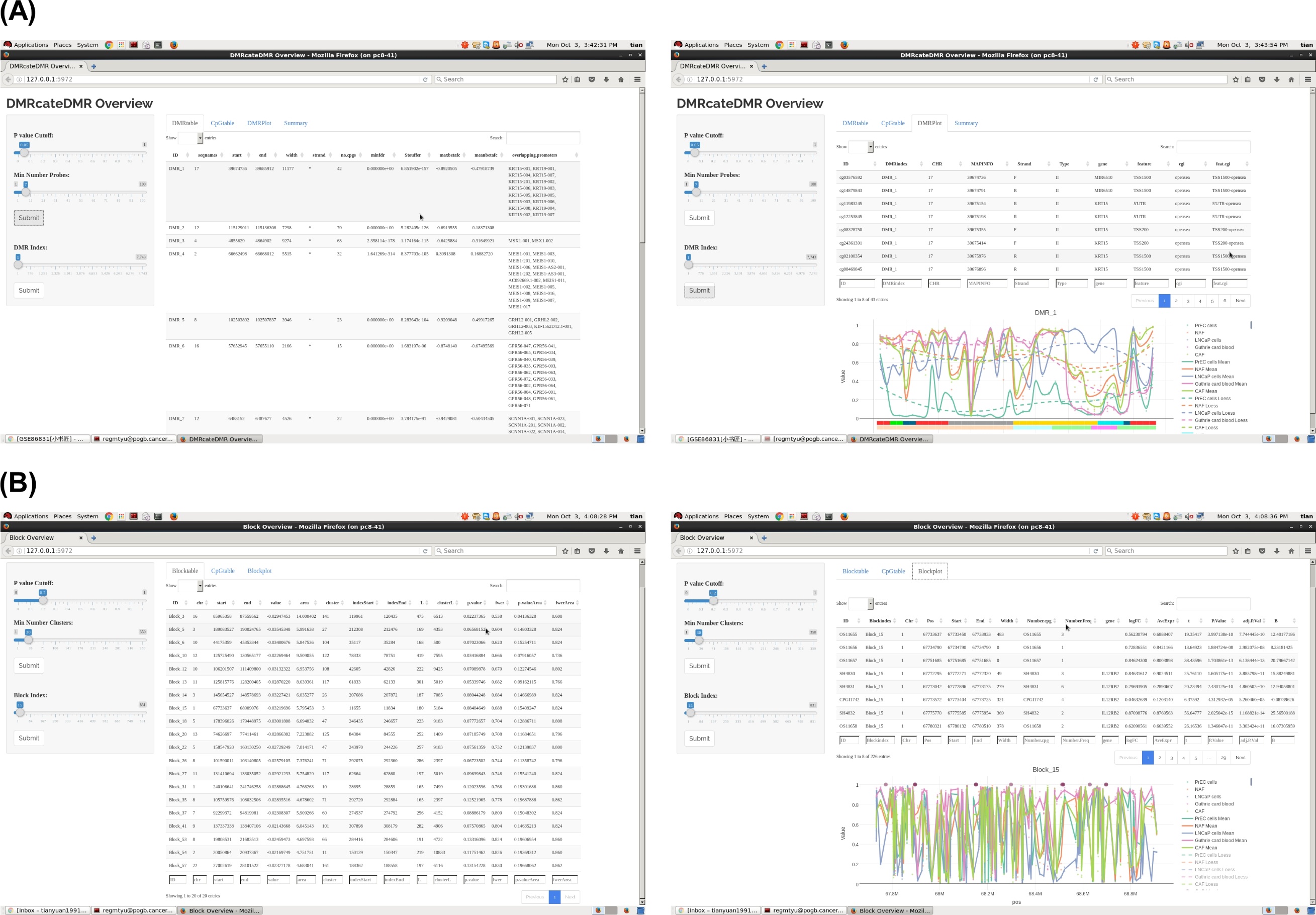


**Figure** 6: DMR and DMB interface plots. **(A)**: Two screenshot for DMR.GUI() function, the left plot shows significant DMR calculated by champ.DMR(), the right plot shows one DMR plotted, for each phenotype, one solid line and one dash line would be draw to represent mean value and loess value. **(B)**: Similar to (A), the interface of Block.GUI() function plots shows information of DMB we generated.

A next step may be to perform GSEA on the called DMPs and DMRs, which could be done with following code:

myGSEA <- champ.GSEA(DMP=myDMP,arraytype = "EPIC")

Just as code above shows, champ.GSEA() function would automatically fetch result of DMP and DMR, mapping significant CpGs to genes, then conduct GSEA on them separately. Users may refer to the vignette and manual for more details. We incorporated “goseq” package into GSEA to correct the bias that some genes contain more CpGs would have more probability get selected as significant genes.

Cell-type heterogeneity presents a common problem to the analysis of DNA methylation data and can be adjusted for with ChAMP. There is one functions provided: champ.refbase() method for reference based cell type proportion calculation and correction. However, refbase method should only works on Blood data set, because currently we only incoperated Whole Blood Reference in it. So this data is not suitable to test refbase method because there are Cell Line samples in it.

As with 450k data, EPIC bead arrays offer the possibility to use methylation signal intensities to infer copy-number states. This is accomplished using the champ.CNA() function. champ.CNA() provides two ways to calculate copy number aberration status, one is comparing each sample to a control phenotype, while the other approach is to compare each sample to the average copy number status across all samples in the study, to see if any sample shows aberrant copy-number variance relative to the average. Here we choose the second approach and calculate the copy number aberration level of each sample relative to the average over all samples:

myCNA <- champ.CNA(control = F,arraytype = "EPIC")

Then we could get copy number aberration plot as below:


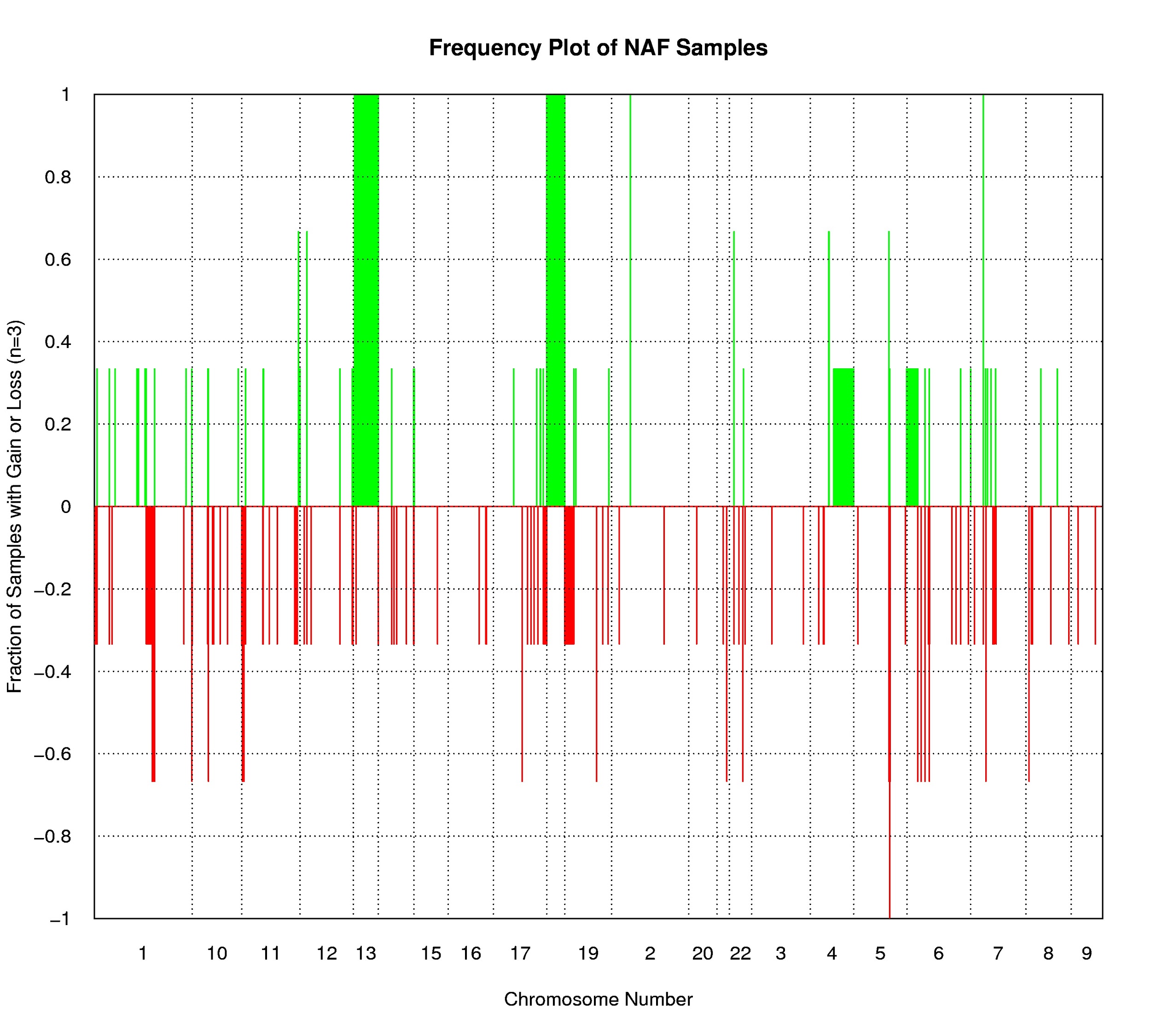


**Figure** 7**:** Copy number aberration for NAF samples, generated from champ.CNA() function.

Finally, we demonstrate the champ.EpiMod() function on this dataset. champ.EpiMod() enables the identification of hotspots (gene modules) of differential methylation in a user-specified gene network, for instance, this could be a protein-protein interaction network. By applying following code, we can identify differential methylated gene modules present within large PPI networks:

myEpiMod <- champ.EpiMod(arraytype="EPIC")

This outputs a PDF file for each identified differential methylated module. Below is the result of this function on a TCGA 450K Endometrial Cancer data, which correctly retrieves an important module centred on the HAND2 gene, which is a causal driver of endometrial cancer.


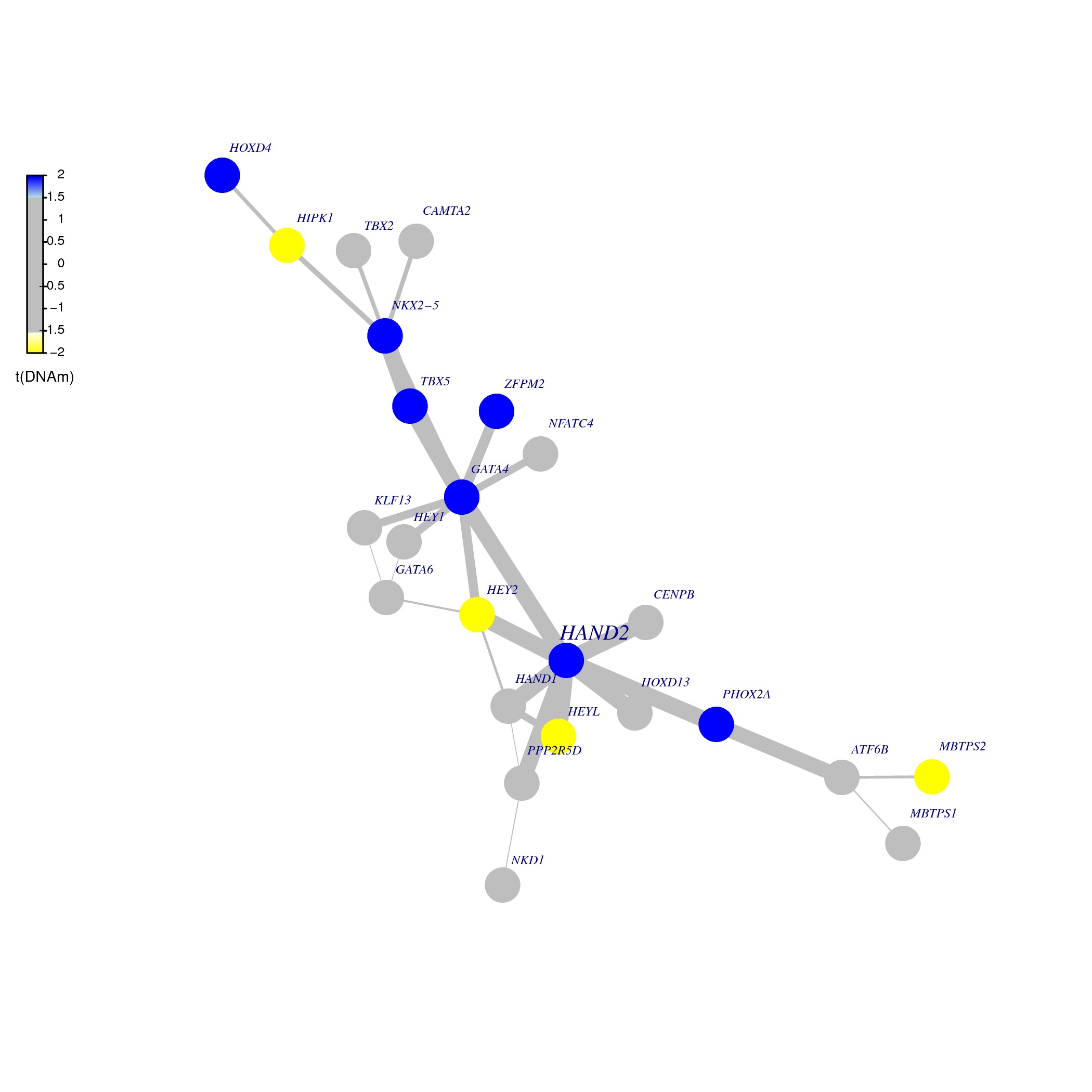


**Figure** 8 Differential Methylated Hotspots calculated from champ.EpiMod on TCGA Endometrial Cancer data. Each node is one gene, with the color indicative of the level of differential DNA methylation between normal and cancer tissue. Edge weights are proportional to the average of the absolute statistics of differential methylation of the two genes that make up the edge.
